# Thermodynamic Properties of Crystalline Cellulose Allomorphs Studied with Dispersion-Corrected Density Functional Methods

**DOI:** 10.3390/molecules27196240

**Published:** 2022-09-22

**Authors:** Divya Srivastava, Jouni Ahopelto, Antti J. Karttunen

**Affiliations:** 1 Department of Chemistry and Materials Science, Aalto University, P.O. Box 16100, FI-00076 Aalto, Finland; 2 VTT Technical Research Centre of Finland Ltd., P.O. Box 1000, FI-02044 Espoo, Finland

**Keywords:** cellulose, thermodynamics, phonon properties, quasiharmonic approximation, density functional theory, quantum chemical calculations

## Abstract

The phonon properties and thermodynamics of four crystalline cellulose allomorphs, Iα, Iβ, II, and III${}_{1}$, have been investigated using dispersion-corrected density functional theory (DFT). In line with experimental findings, the free energy differences between the studied cellulose allomorphs are small, less than 1 kJ/mol per atom. The calculated specific heat at constant volume ($C_{v}$) has been compared with the available experimental data in the temperature range 10–300 K. Quasiharmonic approximation has been employed to study thermodynamics and specific heat at constant pressure ($C_{p}$). For the studied temperature range of 10–400 K, the specific heat of all cellulose allomorphs shows very similar behavior. The calculated and experimental specific heat agree well at low temperatures below 100 K, but the deviation between theory and experiment increases with temperature. This may be due to increasing phonon anharmonicity as the temperature increases.

## 1. Introduction

**Figure 1:**
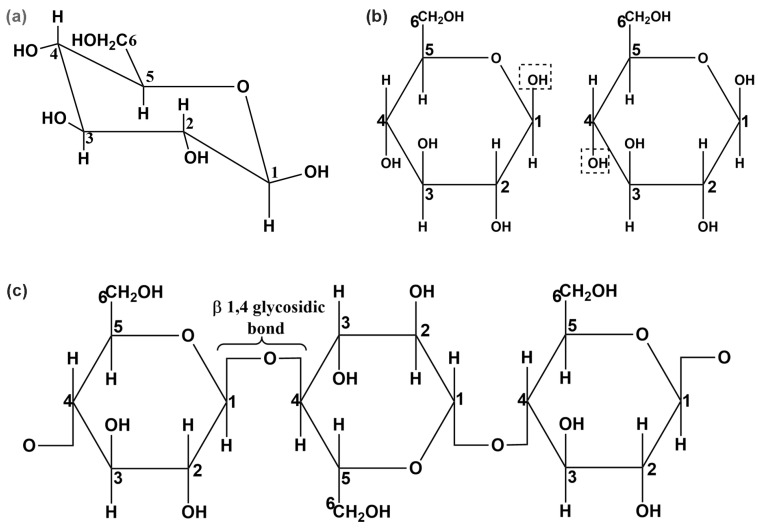
(**a**) Chair conformation of β-D-glucose. (**b**) Two OH groups (inside the dotted boxes) are far apart and do not form a covalent 1,4 glycosysdic bond. (**c**) Chemical structure of cellulose (β-D-glucose in Haworth projection).

Cellulose is the most abundant organic material on Earth. It is a linear polysaccharide, made up of D-glucopyranose rings. Thousands of D-glucose rings in cellulose are joined together via β1→4 glycosidic bonds. The repeating D-glucose rings in cellulose possess the thermodynamically preferred ^4^C_1_ conformation, with all three hydroxyl groups in an equatorial position and all hydrogen atoms in axial positions, as depicted in Figure 1a [1].

In cellulose, D-glucopyranose molecules have β orientation; therefore, the hydroxyl group (−OH) of anomeric carbon 1 (C^1^) and the hydroxymethyl group (−CH_2_OH) of carbon 5 (C^5^) are on the same face of D-glucopyranose rings and directed above the plane of the rings (see Figure 1b). The hydroxyl groups on C^1^ and carbon 4 (C^4^) are in opposite directions. The hydroxyl groups on C^1^ and C^4^ of two glucose molecules are far from each other and unable to form a β1→4 glycosidic bond. The only way that β-D-glucose molecules can join together and form a polymer is if alternate β-D-glucose molecules are inverted. When β-D-glucose molecules are joined together via a β1→4 glycosidic bond, they form a linear chain of cellulose (no coiling or branching), as shown in Figure 1c.

The linear chains of cellulose are arranged parallel to each other through hydrogen bonding and weak van der Waals interactions. Equatorial hydroxyl groups form hydrogen bonds with their nearest neighbors, which allows cellulose to have a crystalline fiber structure and morphology. X-ray diffraction analysis shows that native cellulose is composed of so-called cellulose I, which consists of two distinct crystal phases classified as I and I [2]. The ratio of cellulose I and cellulose I depends on the origin of the cellulose.

Cellulose I has a triclinic crystal structure (space group P1) and its unit cell contains one cellulose chain (42 atoms) [3]. For comparison, cellulose I is monoclinic (space group $P2_{1}$), with two conformationally distinct parallel chains per unit cell (84 atoms) [4].

**Figure 2:**
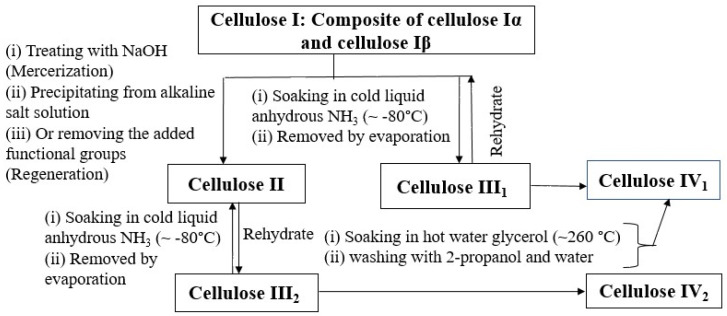
Illustration of interconversion of cellulose I and cellulose II into other cellulose allomorphs [5,6,7,8].

Apart from native cellulose I phases, cellulose exists in several different crystal forms (allomorphs), namely cellulose II, III${}_{1}$, III${}_{2}$, IV${}_{1}$, and IV${}_{2}$, which can be synthesized by the thermochemical treatment of cellulose. The steps involved in the interconversion of different cellulose allomorphs are shown in Figure 2.

Cellulose II is synthesized from cellulose I [7] and has a monoclinic crystal structure (space group $P2_{1}$). Its unit cell contains two cellulose chains, which are arranged in an antiparallel manner [9]. Cellulose III${}_{1}$ and III${}_{2}$ are prepared from cellulose I and II, respectively. Cellulose III${}_{1}$ has a monoclinic unit cell, containing one cellulose chain. In cellulose III${}_{1}$, chains are arranged in parallel fashion, as in cellulose I, with slightly different conformations [10,11].

The crystal structure of cellulose III${}_{2}$ is not well established yet. Both cellulose III${}_{1}$ and III${}_{2}$ revert back to their original forms when they are treated with boiling water.

Cellulose IV${}_{1}$ and IV${}_{2}$ are prepared from cellulose III${}_{1}$ and III${}_{2}$, respectively, by thermal treatment in hot glycerol. Gradiner and Sarko [6] proposed that cellulose IV${}_{1}$ has an orthorhombic unit cell with two cellulose chains. It was also reported that cellulose IV${}_{1}$ and IV${}_{2}$ have unit cells of almost the same size, with a different arrangement of cellulose chains, parallel for cellulose IV${}_{1}$ and antiparallel for cellulose IV${}_{2}$. However, the crystal structure of cellulose IV has not yet been determined with X-ray crystallography and its space group is undefined.

The thermodynamic properties of crystalline and amorphous cellulose allomorphs have been studied previously, both experimentally and computationally. Goldberg et al. studied the thermodynamics of crystalline cellulose allomorphs Iβ, II, and III${}_{1}$, and concluded that it was not possible to determine the relative stabilities of the allomorphs with a reasonable degree of certainty [12]. Dri et al. used dispersion-corrected density functional methods to study the anisotropy and temperature dependence of the structural, thermodynamic, and elastic properties of crystalline cellulose Iβ [13]. ReaxFF molecular dynamics simulations have been used to study the phonon properties of cellulose Iβ [14] and the phonon transport in cellulose nanocrystals [15]. However, the phonon properties and thermodynamics of all crystalline cellulose allomorphs have not been studied systematically with first-principles electronic structure methods.

Our aim is to provide a better understanding of the thermodynamic properties of four crystalline cellulose allomorphs: Iα, Iβ, II, and III${}_{1}$. To accomplish this, we employ quantum chemical methods to study the crystal structure, vibrational properties, and thermodynamic properties such as free energy, heat capacity, and entropy of cellulose Iα, Iβ, II, and III${}_{1}$. Where experimental data are available, we compare our computational results systematically with experimental findings.

## 2. Computational Methods

We carried out periodic density functional theory (DFT) calculations using the DFT-PBE generalized gradient approximation (GGA) exchange-correlation functional [16]. All calculations were performed using the CRYSTAL17 program package [17]. We used the Gaussian-type triple-ζ-valence plus polarization level basis set (TZVP), derived from the molecular Karlsruhe def2 basis set [18]. The used basis sets are the same as in Ref. [19]. Weak van der Waals-type dispersion interactions were described using Grimme’s semi-empirical DFT-D3 dispersion correction scheme with zero-damping (ZD) [20,21]. All calculations reported here were performed at the DFT-PBE-D3(ZD)/TZVP level of theory. The use of the dispersion-corrected DFT method is important to account for the weak interactions between the cellulose sheets.

The geometries of the studied bulk cellulose allomorphs were fully optimized within their respective space groups. The default CRYSTAL17 convergence criteria were used in the geometry optimizations. Coulomb and exchange integral tolerances were set to tight values of 8, 8, 8, 8, and 16. The reciprocal space was sampled with Monkhorst–Pack *k*-meshes, which are reported in Table S1 in the Supplementary Materials [22]. Thermal properties were investigated using quasi-harmonic approximation (QHA), as implemented in CRYSTAL code [23,24,25,26]. The QHA calculations were carried out for the primitive cells of the cellulose allomorphs.

Phonopy was used to perform harmonic phonon calculations with the finite displacement method [27,28]. For the phonon calculations, the following phonon supercells were used: 2×3×2 for cellulose Iα, 2×2×2 for cellulose Iβ, 2×2×2 for cellulose II, and 5×2×3 for cellulose III${}_{1}$. The default displacement of 0.01 Å was used. The phonon calculations showed the following small imaginary frequencies around the Γ point: 2.7*i* cm${}^{-1}$ for Iα, 7.1*i* cm${}^{-1}$ for Iβ, 6.8*i* cm${}^{-1}$ for II, and 4.7*i* cm${}^{-1}$ for III${}_{1}$. These modes persisted even when the supercells were further extended. They can arise, for example, from small inaccuracies in the numerical integration of the exchange-correlation functional. The Brillouin zone paths for the phonon dispersion relations were obtained from the SeeK-path web service [29]. Phonon density of states (DOS) was calculated with the linear tetrahedron method. Atom-projected phonon density of states (PDOS) was calculated using Gaussian smearing to reduce memory requirements. The Monkhorst–Pack type *q*-meshes used for phonon calculations are given in Table S1 of the Supplementary Materials. Thermodynamic properties were calculated with the help of the harmonic phonon dispersion relations.

## 3. Results

### 3.1. Geometry Optimization and Thermodynamics

First, we discuss the crystalline structure of cellulose allomorphs, optimized at the DFT-PBE-D3(ZD)/TZVP level of theory. The optimized lattice parameters for all studied cellulose allomorphs are given in Table 1. For all cases, cellulose chains form hydrogen-bonded cellulose sheets along the *a* direction, the *b* axis is the chain axis with covalent bonds, and the *c* axis is the stacking direction of cellulose sheets. The optimized lattice parameters are in reasonable agreement with the experimental lattice parameters. The deviation of the optimized lattice parameter *c* from the experimental values is 2%, 3.7%, 6.9%, and 2.5%, for cellulose Iα, Iβ, II, and III${}_{1}$, respectively. The deviation of optimized lattice parameter *b* from the experimental values is 2.7%, 1.1%, 2.8%, and 3.4% for cellulose Iα, Iβ, II, and III${}_{1}$, respectively. In the case of cellulose Iα and Iβ, there are no strong inter-sheet O-H⋯O hydrogen bonds and sheets are stacked together through van der Waals interactions and weak inter-sheet C-H⋯O interactions. In cellulose II and III, the cellulose sheets are stacked together via hydrogen bonds. In cellulose II, O2-H⋯O6 or O6-H⋯O2 hydrogen-bonded sheets are stacked through hydrogen bonds formed between O6 atoms of the anti-parallel chain. This type of hydrogen bonding is absent in cellulose III${}_{1}$. In cellulose III${}_{1}$, O2-H⋯O6 hydrogen-bonded sheets are stacked through O6-H⋯O2 inter-sheet hydrogen bonds [10].

Compared to the previous quantum chemical studies on cellulose allomorphs [19], the DFT-D3 dispersion correction is slightly less successful than DFT-D2 dispersion correction [30] in describing the weak dispersion interactions in cellulose allomorphs. In particular, the *c* parameter of cellulose II is underestimated by 6.9% here, while the difference from the experimental lattice parameter was less than 1.0% in our previous study. However, the obtained relative energies are in line with the previous results (see below), and in the present study, we chose to use DFT-D3 as the QHA calculations in CRYSTAL17 do not work in conjunction with DFT-D2.

We studied the energetics and thermodynamics of the cellulose allomorphs by comparing their electronic energies (*E*), Gibbs free energies (*G*, constant pressure and temperature), and Helmholtz free energies (*F*, constant volume and temperature). We define relative (free) energy ΔX for each allomorph with respect to the free energy of cellulose Iβ as:

ΔX=X(allomorph)/Z(allomorph)−X(celluloseIβ)/Z(celluloseIβ),

where *X* is electronic energy *E*, the Gibbs free energy for the primitive cell is $G_{\Gamma }$, the quasi-harmonic Helmholtz free energy for the primitive cell is $F_{QHA,\Gamma }$, and the harmonic Helmholtz free energy obtained from phonon supercell calculations is $F_{Harm,SC}$. Z(allomorph) is the number of cellulose chains in the unit cell.

At 300 K, cellulose Iβ is the energetically most favorable cellulose allomorph, followed by cellulose Iα and cellulose III${}_{1}$ (Table 1). The energy differences between the allomorphs are not large: 27.5 kJ/mol per *Z* means only 0.7 kJ/mol per atom, which is a small energy difference. $\Delta G_{\Gamma }$ and $\Delta F_{QHA,\Gamma }$ are almost identical, suggesting that taking quasi-harmonic approximation into account does not have a significant effect on the thermodynamic considerations. When comparing *E* and $\Delta G_{\Gamma }$, it appears that entropy favors cellulose Iβ over cellulose Iα, while cellulose II and III${}_{1}$ are entropically slightly favored in comparison to cellulose Iβ. Finally, the quantity $\Delta F_{Harm,SC}$ includes more complete sampling of the phonon frequencies and represents the best estimate of the thermodynamics of the cellulose allomorphs for the present study. Cellulose Iβ remains the thermodynamically favored allomorph, followed closely by cellulose Iα and III${}_{1}$. Cellulose II is thermodynamically the least favorable allomorph based on $\Delta F_{Harm,SC}$, but the free energy difference from cellulose Iβ is only 0.6 kJ/mol per atom. This is in line with an experimental study on the thermodynamics of cellulose allomorphs Iβ, II, and III${}_{1}$, which concluded that it was not possible to determine the relative stabilities of the allomorphs with a reasonable degree of certainty [12]. We note that our findings consider only the thermodynamics of the cellulose allomorphs, and kinetics and energy barriers related to the interconversion processes between the cellulose allomorphs have not been considered.

### 3.2. Phonon Dispersion Relations

**Figure 3:**
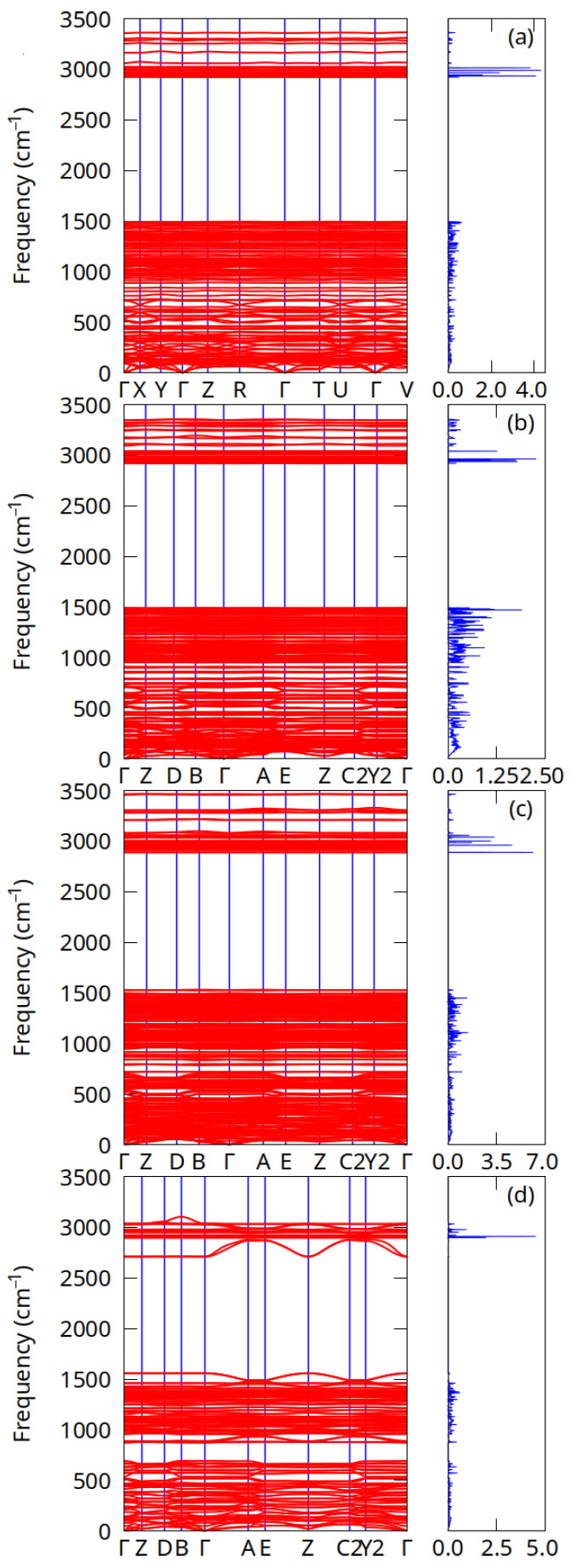
Harmonic phonon dispersion relations along high symmetry points (**left**) and phonon density of states (**right**): (**a**) cellulose Iα, (**b**) cellulose Iβ, (**c**) cellulose II, and (**d**) cellulose III${}_{1}$.

The phonon dispersion relations for cellulose Iα, Iβ, II, and III${}_{1}$, together with the phonon density of states, are illustrated in Figure 3. The unit cells of cellulose Iα, Iβ, II, and III${}_{1}$ contain 42, 84, 84, and 42 atoms respectively, giving rise to 126, 252, 252, and 126 phonon modes at each phonon wave-vector. Out of these modes, three are acoustic and the rest are optical modes. Due to the relatively large unit cells, these systems have rather complex phonon dispersion relations. A zoomed-in phonon dispersion plot in the low-frequency region up to 500 cm${}^{-1}$ is shown in Figure S1 in the Supplementary Materials. The phonon frequencies span a range of approximately 0–3500 cm${}^{-1}$ for these systems. Acoustic and optical modes cross in each case, meaning that these systems show no phonon band gap. However, there is a large gap between optical modes at around 1500 and 3000 cm${}^{-1}$. The acoustic modes and low-frequency optical branches show some dispersion, but high-frequency optical modes above 3000 cm${}^{-1}$ are nearly flat. These dispersionless optical modes suggest that their group velocities are nearly zero and they behave as standing waves. The atom-projected density of states for the cellulose allomorphs is shown in Figure S2 of the Supplementary Materials. The high-frequency optical branches (>3000 cm${}^{-1}$) correspond to C–H and O–H stretching vibrations, while the optical modes 500 to 1500 cm${}^{-1}$ correspond to, for example, bending modes (a more detailed assignment of vibrational spectra is available in Ref. [19]).

### 3.3. Specific Heat Capacity and Gruneisen Parameter

**Figure 4:**
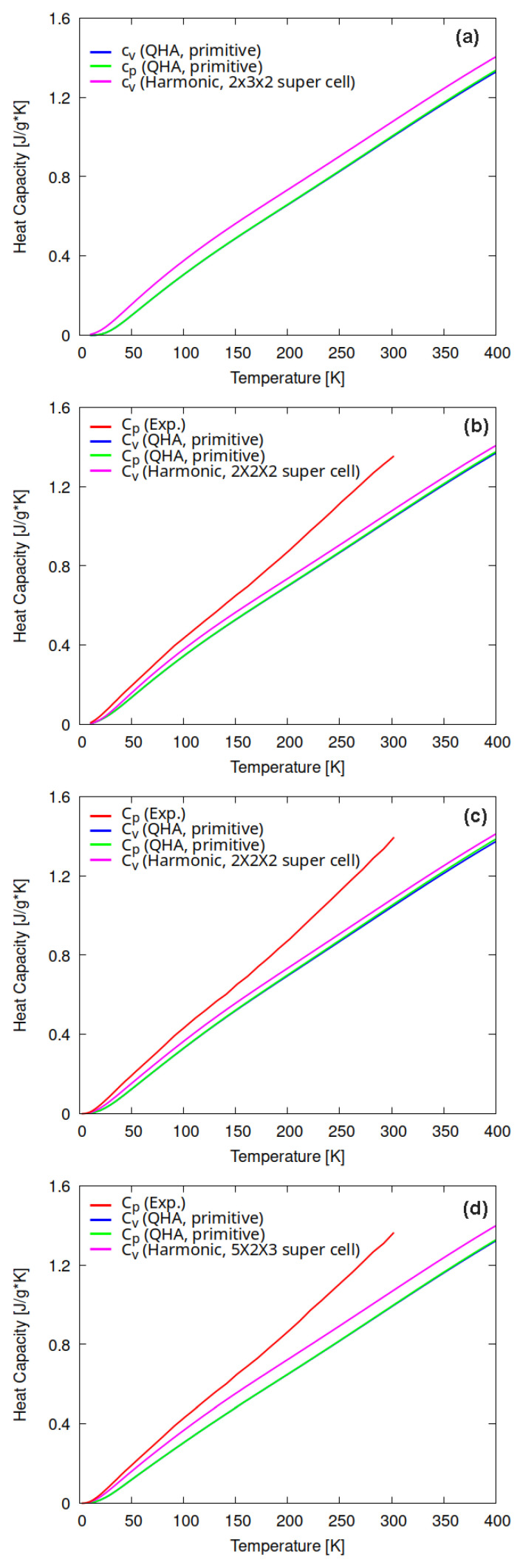
Temperature dependence of specific heat capacity at constant volume ($C_{v}$) and constant pressure ($C_{p}$), computed using harmonic and quasi-harmonic approximation for (**a**) cellulose Iα, (**b**) cellulose Iβ, (**c**) cellulose II, and (**d**) cellulose III${}_{1}$. Available experimental data are also plotted [12].

Based on the phonon dispersion relations, we investigated the specific heat capacity of the cellulose allomorphs. Specific heat capacity is the amount of heat energy per unit mass required to raise the temperature of the material by one degree Kelvin (units used in this study: J/(g·K)). The specific heat capacity at constant volume ($C_{v}$) was calculated using the harmonic phonon dispersion relations shown in Figure 3. The $C_{v}$ can be effectively calculated within the harmonic approximation. However, other thermodynamic properties, such as specific heat capacity at constant pressure, $C_{p}$, thermal expansion, and the temperature dependence of the bulk modulus, cannot be computed using the harmonic approximation. In this respect, quasi-harmonic approximation (QHA) is a simple and powerful approach that provides volume-dependent phonon frequencies. The QHA calculations were carried out for 10–400 K and at 1 atm pressure, using the primitive cell of each cellulose allomorph. Figure 4 shows the specific heat capacity at constant volume $C_{v}$ obtained from the harmonic and quasi-harmonic approximations. For all systems, harmonic and quasi-harmonic $C_{v}$ are qualitatively consistent and $C_{v}$ increases similarly with temperature. The $C_{v}$ obtained with harmonic approximation and phonon supercells differ only slightly from the results obtained with the QHA approach using the primitive cell only. The calculated specific heat capacity at constant volume ($C_{v}$) and the specific heat capacity at constant pressure ($C_{p}$) were also compared with the available experimental values [12]. The experimental data in Ref. [12] were obtained for commercially available cellulose Iβ and its derivatives. As the difference between QHA $C_{p}$ and QHA $C_{v}$ is insignificant and they increase monotonously with the temperature, it is reasonable to compare the calculated harmonic $C_{v}$ with experimental $C_{p}$. The harmonic $C_{v}$ values obtained from phonon supercell calculations are slightly closer to the experimental values compared to QHA values obtained for the primitive cell. The computational results are in good agreement with the experimental data for low temperatures. In each case, the difference between theory and experiments increases as the temperature increases. A similar trend has been obtained in previous computational studies using ReaxFF molecular dynamics or first-principles DFT methods [14]. The difference between the experimental $C_{p}$ and calculated harmonic $C_{v}$ is somewhat smaller for cellulose III${}_{1}$ compared to cellulose Iβ and II. This might be related to the fact that, in the case of cellulose III${}_{1}$, it was possible to use a slightly larger phonon supercell.

**Figure 5:**
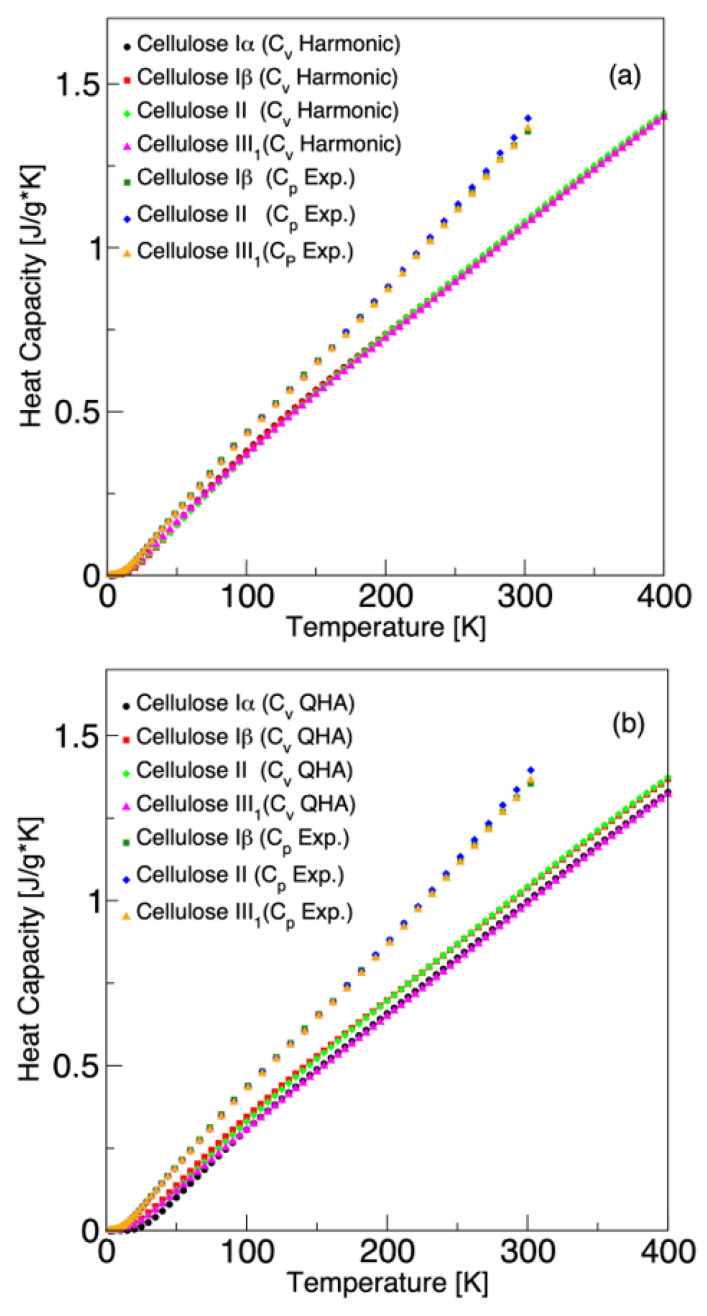
Temperature dependence of thermodynamic properties of cellulose allomorphs. (**a**) Specific heat capacity $C_{v}$ obtained with harmonic approximation. (**b**) Specific heat capacity $C_{v}$ obtained from quasi-harmonic approximation. Experimental $C_{p}$ is included for comparison [12].

Figure 5a,b compare the $C_{v}$ of cellulose Iα, Iβ, II, and III${}_{1}$, obtained from the harmonic and quasi-harmonic approximation, with available experimental $C_{p}$ data for cellulose Iβ, II, and III${}_{1}$. For all cases, the specific heat capacity is plotted as a function of the temperature. It can be clearly seen that the specific heat capacity values lie almost on top of each other for all cellulose allomorphs. Therefore, the specific heat capacity as a function of temperature for any type of crystalline cellulose shows qualitatively and even quantitatively a universal behavior in the temperature range 10–400 K.

**Figure 6:**
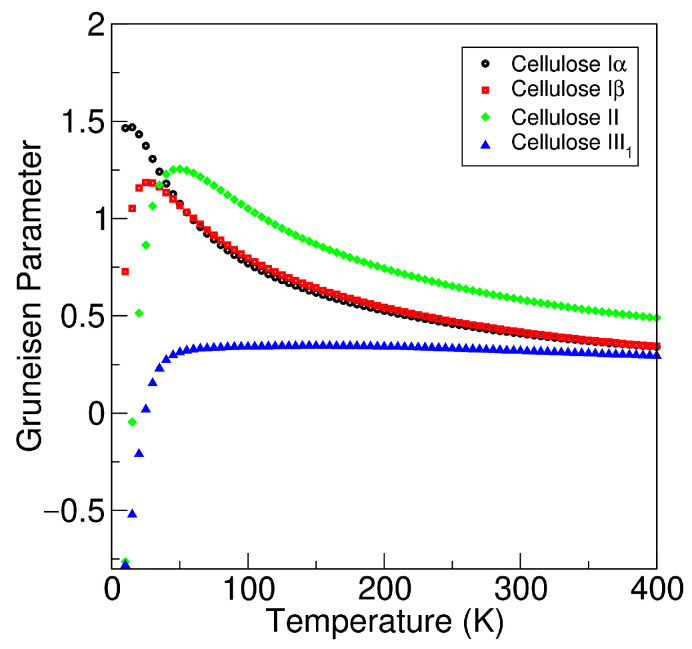
Gruneisen parameter plotted as a function of temperature for cellulose allomorphs Iα, Iβ, II, and III${}_{1}$.

The thermodynamic Gruneisen parameters as a function of temperature for cellulose Iα, Iβ, II, and III${}_{1}$ are shown in Figure 6. The Gruneisen parameter can be considered as a measure of anharmonicity. At low temperatures, there are larger differences between the cellulose allomorphs, but the values converge as the temperature increases above 200 K. At *T* = 300 K, the calculated value of the dimensionless Gruneisen parameter is 0.41 for cellulose Iα, 0.42 for cellulose Iβ, 0.58 for cellulose II, and 0.32 for cellulose III${}_{1}$. The values are rather close to each other, but cellulose II clearly shows the largest Gruneisen parameter at room temperature. Overall, the absolute values of the Grüneisen parameter are relatively small, suggesting that there is not any anomalous phonon anharmonicity related to the cellulose allomorphs.

## 4. Conclusions

The phonon properties and thermodynamics of cellulose Iα, Iβ, II, and III${}_{1}$ have been studied with the dispersion-corrected density functional method and quasi-harmonic approximation. Cellulose Iβ is the energetically and thermodynamically most favorable allomorph, but the free energy differences between the allomorphs are rather small. The phonon dispersion relations of all studied allomorphs were calculated and the specific heat capacities at both constant volume ($C_{v}$) and constant pressure ($C_{p}$) were studied. The quasi-harmonic approach (QHA) has been employed to compute $C_{p}$. For all studied cellulose allomorphs, $C_{p}$ and $C_{v}$ are rather similar. Furthermore, the $C_{v}$ and $C_{p}$ of different allomorphs are very close to each other. The calculated and experimental $C_{v}$ and $C_{p}$ agree well at low temperatures below 100 K, but the deviation between theory and experiment increases with temperature. This may be due to the increasing phonon anharmonicity as the temperature increases. Furthermore, the present computational results have been obtained for ideal single crystals, while, in the case of the experimental studies, the samples can have a significant amount of surface and various surface effects may affect the thermodynamic properties. Future studies on the phonon anharmonicity of cellulose allomorphs should clarify the remaining discrepancies between theory and experiments. The role of water in the thermodynamics of various cellulose allomorphs and their surfaces should also be investigated, for example, by means of abinitio molecular dynamics.

## Figures and Tables

**Table 1:** Optimized lattice parameters and relative energies (ΔE, ΔG, and ΔF in units of of kJ/mol per *Z*) of crystalline cellulose allomorphs Iα, Iβ, II, and III${}_{1}$ at the DFT-PBE-D3(ZD)/TZVP level of theory (*T* = 300 K for the free energies). The corresponding experimental lattice parameters are given in parentheses, with experimental references given in the header row.

	Iα [3]	Iβ [4]	II [9]	III${}_{1}$ [10]
Crystal system	Triclinic	Monoclinic	Monoclinic	Monoclinic
Space group	P1	$P2_{1}$	$P2_{1}$	$P2_{1}$
*a* (Å)	6.54 (6.72)	8.11 (8.20)	7.88 (8.10)	7.59 (7.85)
*b* (Å)	10.39 (10.40)	10.40 (10.38)	10.45 (10.31)	10.34 (10.31)
*c* (Å)	5.84 (5.96)	7.50 (7.78)	8.45 (9.03)	4.34 (4.45)
α(°)	117.7 (118.1)	90	90	90
β(°)	114.9 (114.8)	95.92 (96.5)	114.11 (117.1)	101.12 (105.1)
γ(°)	81.6 (80.4)	90	90	90
*Z*	1	2	2	1
ΔE	6.6	0	27.5	14.0
$\Delta G_{\Gamma }$	10.5	0	25.8	10.3
$\Delta F_{QHA,\Gamma }$	10.7	0	25.5	11.3
$\Delta F_{Harm,SC}$	5.3	0	24.5	7.67

## Data Availability

Optimized geometries of the studied systems are available in the Supplementary Materials.

## Supplementary Materials

The following supporting information can be downloaded at: https://www.mdpi.com/article/10.3390/molecules27196240/s1. Figure S1: Harmonic phonon dispersion relations in the low-frequency region up to 500 cm${}^{-1}$; Figure S2: Atom-projected phonon density of states; Table S1: Monkhorst–Pack q-meshes used in the calculations; Optimized geometries of the studied structures in CRYSTAL input format.
