# Supplementary material for: Thermodynamic Properties of Crystalline Cellulose Allomorphs Studied with Dispersion-Corrected Density Functional Methods
Source: Molecules. 2022 Sep 22;27(19):6240. doi: 10.3390/molecules27196240 (PMC9570836; doi:10.3390/molecules27196240)
Supplement: Supplementary file 1 [file molecules-27-06240-s001.zip › molecules-1903662-supplementary.pdf]

Supporting Information for "Thermodynamic properties of crystalline cellulose allomorphs studied with dispersion-corrected density functional methods"

Divya Srivastava and Antti J. Karttunen*

Department of Chemistry and Materials Science,

Aalto University, P.O. Box 16100, FI-00076 Aalto, Finland

Jouni Ahopelto

VTT Technical Research Centre of Finland Ltd,

P.O. Box 1000, FI-02044 VTT, Espoo, Finland

* antti.karttunen@aalto.fi

I. SUPPORTING FIGURES

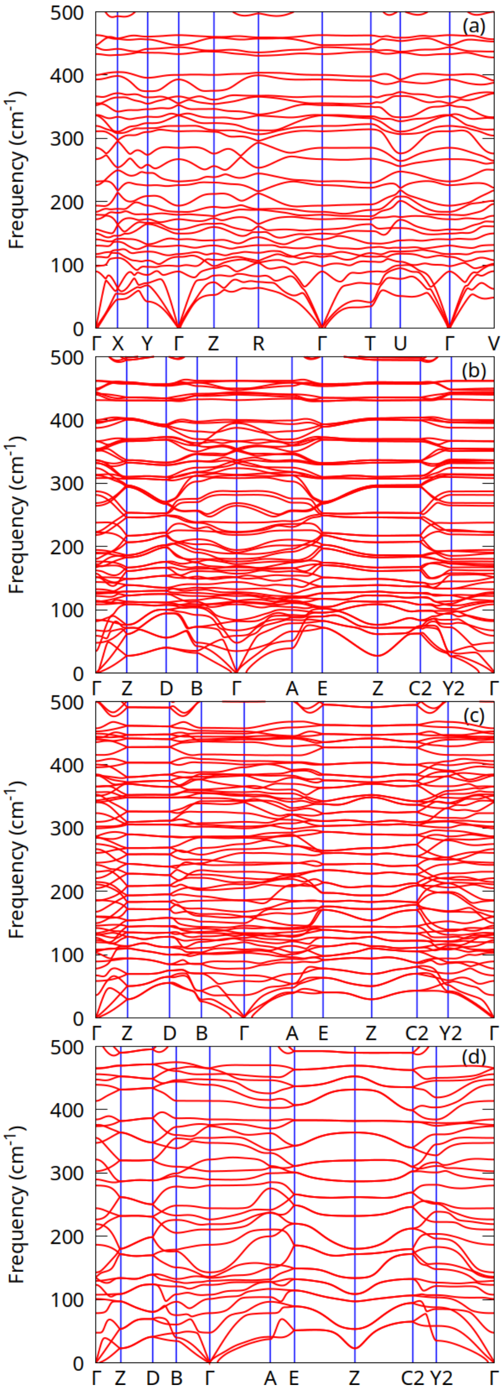

Figure S1. Harmonic phonon dispersion relations in the low-frequency region up to 500 cm^{-1}. (a) Cellulose Iα, (b) cellulose Iβ, (c) cellulose II, and (d) cellulose III_1

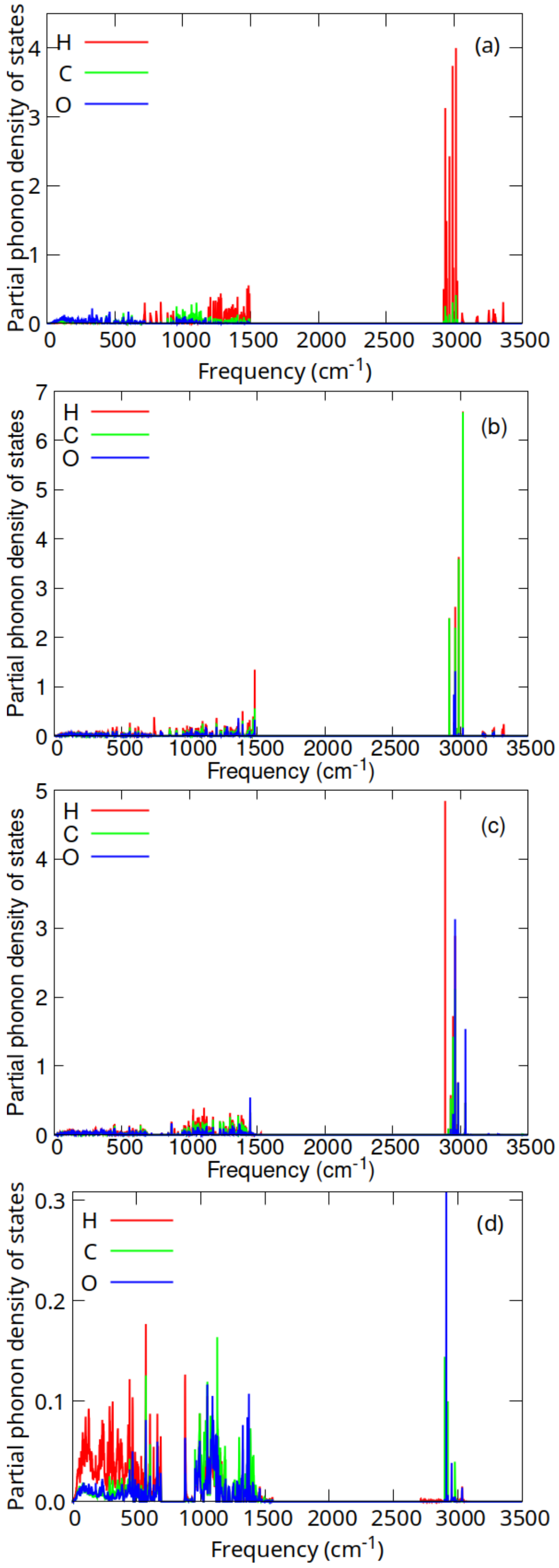

Figure S2. Atom-projected phonon density of states for (a) cellulose Iα, (b) cellulose Iβ, (c) cellulose II and (d) cellulose III$_1$

II. ADDITIONAL COMPUTATIONAL DETAILS

Table S1. Monkhorst-Pack **q**-meshes used in the geometry optimizations, for the evaluation of ther-modynamic properties, phonon DOS, and phonon partial DOS.

Allomorph	Optimization	Thermodynamics	Phonon DOS	Phonon PDOS
Iα	$4 \times 3 \times 4$	$40 \times 40 \times 30$	$20 \times 32 \times 35$	$20 \times 32 \times 35$
Iβ	$3 \times 3 \times 3$	$30 \times 30 \times 30$	$30 \times 30 \times 30$	$26 \times 20 \times 26$
II	$3 \times 3 \times 3$	$30 \times 30 \times 30$	$30 \times 30 \times 30$	$26 \times 20 \times 23$
III_1	$5 \times 3 \times 3$	$50 \times 30 \times 30$	$50 \times 30 \times 30$	$47 \times 20 \times 27$

III. OPTIMIZED GEOMETRIES OF THE STUDIED STRUCTURES

Lattice parameters and atomic positions of the studied structures are given below in CRYSTAL input format (DFT-PBE-D3(ZD)/TZVP level of theory)

Cellulose Iα

```
CRYSTAL
1 0 0
P1
6.54 10.39 5.84 117.67 81.60 114.94
42
1   -2.461238650154E-01    -1.483043831064E-01      2.243414209314E-01
1   -3.496391741872E-01     3.057110111942E-01      3.611220840702E-01
1    3.455908683702E-01    -1.951450338435E-01     -3.483374912620E-01
1   -4.948478820721E-01    -4.349624193740E-02      4.437053126895E-01
1   -4.850740509085E-01     4.909667335888E-01     -4.156908470526E-01
1    2.489793800272E-01     3.738323411812E-01     -1.983182769469E-01
8    1.519781861312E-01     2.385905282064E-01     -5.899220183521E-02
6   -5.808140198194E-02     2.532532057278E-01      3.093379857585E-02
1   -1.789247533749E-01     1.904388663296E-01     -1.387906463591E-01
8   -1.095204170293E-02     4.143638238530E-01      1.594084067213E-01
6   -1.532157843268E-01     1.864576980647E-01      2.273612189352E-01
1   -2.716481348991E-02     2.589559145030E-01      3.942087587531E-01
6   -1.806752027954E-01     1.472051273705E-02      1.063276028396E-01
1   -3.150751573560E-01    -6.024149667548E-02     -5.125070346729E-02
6    4.110656135627E-02     2.578758434601E-03     -3.910726245964E-03
1    1.710962113159E-01     6.468548272084E-02      1.561777433121E-01
6    1.295556349273E-01     7.628630263144E-02     -1.922196742738E-01
1    1.136346016647E-02     9.323008290143E-03     -3.649464317311E-01
6    3.665735315165E-01     8.725594457821E-02     -2.726695087877E-01
1    4.695043264188E-01     1.314112216578E-01     -9.718358108381E-02
1    4.381676235732E-01     1.731768075624E-01     -3.532594084637E-01
8   -3.660072232431E-01     1.938053367064E-01      2.998454166764E-01
8   -2.446819649646E-01    -4.015798134915E-02      3.025460410885E-01
8    3.621812851183E-01    -6.309869973500E-02     -4.648936495722E-01
8   -1.559815451094E-01    -3.087736564402E-01      7.083466346757E-02
6    5.575674829288E-02    -2.285653198674E-01     -1.743547836464E-02
1    1.765320128839E-01    -1.379791828768E-01      1.518703104554E-01
8    9.105084698044E-03    -1.603229106262E-01     -1.484471945625E-01
6    1.547857175297E-01    -3.455561958700E-01     -2.100988511722E-01
1    3.306622042216E-02    -4.298045585462E-01     -3.794488329085E-01
6    1.846458926189E-01    -4.362563877474E-01     -8.396774621056E-02
1    3.107991900217E-01    -3.525354917665E-01      8.212390709319E-02
6   -4.066884014357E-02     4.873294548712E-01      1.733243197373E-02
1   -1.648621976388E-01     3.974764891387E-01     -1.489083993954E-01
6   -1.325900003477E-01    -3.892400766854E-01      2.046782943519E-01
1   -1.477838516846E-02    -3.035617775573E-01      3.770152082519E-01
```

6	-3.676850692503E-01	-4.615573287062E-01	2.899258134833E-01
1	-4.812314319670E-01	4.415553649410E-01	1.188446499759E-01
1	-4.343156651012E-01	-3.690213706644E-01	3.850737921379E-01
8	3.671351342852E-01	-2.604908473407E-01	-2.796702837179E-01
8	2.595384353383E-01	4.453507367253E-01	-2.712201351609E-01
8	-3.586850548649E-01	4.830833976979E-01	4.693831927923E-01

Cellulose Iβ

```
CRYSTAL
1 0 0
P 1 21 1
8.11 10.40 7.50 95.92
84
6  3.862574494306E-02   -4.044136259429E-02    -1.971975297972E-02
6 -3.862574494306E-02    4.595586374057E-01     1.971975297972E-02
1  7.532806220806E-03   -5.438288918160E-02    -1.656684478878E-01
1 -7.532806220806E-03    4.456171108184E-01     1.656684478878E-01
6  1.848177162538E-01    5.269026343528E-02     1.750627373028E-02
6 -1.848177162538E-01   -4.473097365647E-01    -1.750627373028E-02
1  2.133921913525E-01    5.832055006317E-02     1.641799273246E-01
1 -2.133921913525E-01   -4.416794499368E-01    -1.641799273246E-01
6  1.345686609862E-01    1.848635988195E-01    -5.545625631096E-02
6 -1.345686609862E-01   -3.151364011805E-01     5.545625631096E-02
1  1.152002184079E-01    1.818712758997E-01    -2.032814690356E-01
1 -1.152002184079E-01   -3.181287241003E-01     2.032814690356E-01
6 -2.677183773325E-02    2.268888839873E-01     1.722084722351E-02
6  2.677183773325E-02   -2.731111160127E-01    -1.722084722351E-02
1 -4.127933296466E-03    2.395520168616E-01     1.640041709549E-01
1  4.127933296466E-03   -2.604479831384E-01    -1.640041709549E-01
6 -1.649822851698E-01    1.271409673506E-01    -2.468833738788E-02
6  1.649822851698E-01   -3.728590326494E-01     2.468833738788E-02
1 -1.918916813178E-01    1.160324634327E-01    -1.712880121675E-01
1  1.918916813178E-01   -3.839675365673E-01     1.712880121675E-01
6 -3.252252830243E-01    1.580351339977E-01     5.660709757959E-02
6  3.252252830243E-01   -3.419648660023E-01    -5.660709757959E-02
1 -2.975088257729E-01    1.821962844556E-01     1.993813534415E-01
1  2.975088257729E-01   -3.178037155444E-01    -1.993813534415E-01
1 -4.035988234142E-01    7.137169410027E-02     4.692855238420E-02
1  4.035988234142E-01   -4.286283058997E-01    -4.692855238420E-02
8  3.222950875986E-01    6.827402513226E-03    -6.714411924509E-02
8 -3.222950875986E-01   -4.931725974868E-01     6.714411924510E-02
1  3.492149136738E-01   -8.152567691167E-02    -2.118190484463E-02
1 -3.492149136738E-01    4.184743230883E-01     2.118190484463E-02
8  2.647497296032E-01    2.746707056824E-01    -1.546252836183E-03
8 -2.647497296032E-01   -2.253292943176E-01     1.546252836183E-03
1  2.247189879006E-01    3.621371381663E-01    -3.648618407985E-02
1 -2.247189879006E-01   -1.378628618337E-01     3.648618407985E-02
8 -8.435266388586E-02    3.447815460542E-01    -6.621244160175E-02
8  8.435266388586E-02   -1.552184539458E-01     6.621244160175E-02
8 -1.074896974911E-01    6.805233594580E-03     5.450356920687E-02
8  1.074896974911E-01   -4.931947664054E-01    -5.450356920687E-02
8 -4.120926305052E-01    2.607974955295E-01    -3.861096442572E-02
8  4.120926305052E-01   -2.392025044705E-01     3.861096442572E-02
1  4.677618443545E-01    2.520751657045E-01    -2.399788841086E-02
```

1	-4.677618443545E-01	-2.479248342955E-01	2.399788841086E-02
6	-4.545178186472E-01	-3.076767535038E-01	4.814867954902E-01
6	4.545178186472E-01	1.923232464962E-01	-4.814867954902E-01
1	-4.585229288289E-01	-3.235632663726E-01	3.353815662270E-01
1	4.585229288289E-01	1.764367336274E-01	-3.353815662270E-01
6	-3.161607023322E-01	-2.137965300121E-01	-4.536488502753E-01
6	3.161607023322E-01	2.862034699879E-01	4.536488502753E-01
1	-3.128407180236E-01	-2.071822489673E-01	-3.061374167323E-01
1	3.128407180236E-01	2.928177510327E-01	3.061374167323E-01
6	-3.559776317877E-01	-8.194894895952E-02	4.654280105584E-01
6	3.559776317877E-01	4.180510510405E-01	-4.654280105584E-01
1	-3.600674851679E-01	-8.875384223529E-02	3.179903694384E-01
1	3.600674851679E-01	4.112461577647E-01	-3.179903694384E-01
6	4.732490526490E-01	-3.931790759448E-02	-4.870961484141E-01
6	-4.732490526490E-01	4.606820924055E-01	4.870961484141E-01
1	4.760874493080E-01	-2.620954300302E-02	-3.407168702492E-01
1	-4.760874493080E-01	4.737904569970E-01	3.407168702492E-01
6	3.386206404234E-01	-1.375127610545E-01	4.500443815973E-01
6	-3.386206404234E-01	3.624872389455E-01	-4.500443815973E-01
1	3.231658312236E-01	-1.424908915241E-01	3.027151986533E-01
1	-3.231658312236E-01	3.575091084759E-01	-3.027151986533E-01
6	1.733609369218E-01	-1.114049758328E-01	-4.773227701172E-01
6	-1.733609369218E-01	3.885950241672E-01	4.773227701172E-01
1	1.984982235912E-01	-9.600744250192E-02	-3.316097990458E-01
1	-1.984982235912E-01	4.039925574981E-01	3.316097990458E-01
1	9.541021009854E-02	-1.971947073757E-01	4.985106215573E-01
1	-9.541021009854E-02	3.028052926243E-01	-4.985106215573E-01
8	-1.635950033636E-01	-2.577892044307E-01	4.937396575726E-01
8	1.635950033636E-01	2.422107955693E-01	-4.937396575726E-01
1	-1.494085040481E-01	-3.511001409165E-01	-4.746439905027E-01
1	1.494085040481E-01	1.488998590835E-01	4.746439905027E-01
8	-2.283065640505E-01	6.094222884483E-03	-4.675115341767E-01
8	2.283065640505E-01	-4.939057771155E-01	4.675115341767E-01
1	-2.682421545374E-01	9.449984805660E-02	-4.967143266121E-01
1	2.682421545374E-01	-4.055001519434E-01	4.967143266121E-01
8	4.246605522840E-01	7.849735610807E-02	4.229520820474E-01
8	-4.246605522840E-01	-4.215026438919E-01	-4.229520820474E-01
8	3.865990866645E-01	-2.617274516842E-01	-4.757380213200E-01
8	-3.865990866645E-01	2.382725483158E-01	4.757380213200E-01
8	8.966731163341E-02	-3.663101538070E-03	4.378153129099E-01
8	-8.966731163341E-02	4.963368984619E-01	-4.378153129099E-01
1	-2.945995871590E-02	-1.214960050319E-02	4.543775632327E-01
1	2.945995871590E-02	4.878503994968E-01	-4.543775632327E-01

Cellulose II

```
CRYSTAL
1 0 0
P 1 21 1
7.88 10.45 8.45 114.11
84
6     4.972278290659E-02   3.871575286516E-01   4.419152429238E-03
6    -4.972278290659E-02  -1.128424713484E-01  -4.419152429238E-03
1     2.418567035757E-02   4.061331322736E-01  -1.324921007942E-01
1    -2.418567035757E-02  -9.386686772641E-02   1.324921007942E-01
6     2.108158382980E-01   2.915223929600E-01   7.887774153616E-02
6    -2.108158382980E-01  -2.084776070400E-01  -7.887774153616E-02
1     2.437746244073E-01   2.749660903409E-01   2.174129703600E-01
1    -2.437746244073E-01  -2.250339096591E-01  -2.174129703600E-01
6     1.499979705460E-01   1.649432087512E-01  -2.105175617970E-02
6    -1.499979705460E-01  -3.350567912488E-01   2.105175617970E-02
1     1.126540543238E-01   1.862604123934E-01  -1.595702570585E-01
1    -1.126540543238E-01  -3.137395876066E-01   1.595702570585E-01
8     3.667238507375E-01   3.434610765971E-01   5.654802279892E-02
8    -3.667238507375E-01  -1.565389234029E-01  -5.654802279892E-02
6    -2.685317921631E-02   1.174885211587E-01  -6.265738130378E-03
6     2.685317921631E-02  -3.825114788413E-01   6.265738130378E-03
1     2.944950251439E-03   1.011610155620E-01   1.316477164083E-01
1    -2.944950251439E-03  -3.988389844380E-01  -1.316477164083E-01
8     2.996738606387E-01   7.768324647908E-02   4.316366733634E-02
8    -2.996738606387E-01  -4.223167535209E-01  -4.316366733634E-02
6    -1.808002757876E-01   2.184877497149E-01  -8.236306835155E-02
6     1.808002757876E-01  -2.815122502851E-01   8.236306835155E-02
1    -2.077178746266E-01   2.339460328819E-01  -2.200357540424E-01
1     2.077178746266E-01  -2.660539671181E-01   2.200357540424E-01
8    -9.274884555458E-02  -1.544958314718E-05  -1.005621057058E-01
8     9.274884555458E-02   4.999845504169E-01   1.005621057058E-01
8    -1.159003618059E-01   3.360021721139E-01   1.388813790018E-02
8     1.159003618059E-01  -1.639978278861E-01  -1.388813790018E-02
6    -3.605798243397E-01   1.789380959432E-01  -7.116260283299E-02
6     3.605798243397E-01  -3.210619040568E-01   7.116260283299E-02
1    -3.901644955094E-01   7.945070465172E-02  -1.162735916787E-01
1     3.901644955094E-01  -4.205492953483E-01   1.162735916787E-01
1    -3.437033836343E-01   1.812114754003E-01   6.471229400188E-02
1     3.437033836343E-01  -3.187885245997E-01  -6.471229400188E-02
8     4.916793132294E-01   2.611748864952E-01  -1.750873916751E-01
8    -4.916793132294E-01  -2.388251135048E-01   1.750873916751E-01
6     5.063966518355E-02  -1.678062746735E-01  -4.778782118322E-01
6    -5.063966518355E-02   3.321937253265E-01   4.778782118322E-01
1     6.467661551409E-02  -1.790367300874E-01  -3.423981318910E-01
1    -6.467661551409E-02   3.209632699126E-01   3.423981318910E-01
6     2.011137871569E-01  -7.842638804136E-02  -4.855238663146E-01
```

6	-2.011137871569E-01	4.215736119586E-01	4.855238663146E-01
1	1.834634477210E-01	-7.593168687660E-02	3.780942645029E-01
1	-1.834634477210E-01	4.240683131234E-01	-3.780942645029E-01
6	1.766611560701E-01	5.833017106331E-02	-4.309025007948E-01
6	-1.766611560701E-01	-4.416698289367E-01	4.309025007948E-01
1	2.147567631872E-01	5.748535590663E-02	-2.897598097226E-01
1	-2.147567631872E-01	-4.425146440934E-01	2.897598097226E-01
8	3.816753550370E-01	-1.285060088532E-01	-3.832998660089E-01
8	-3.816753550370E-01	3.714939911468E-01	3.832998660089E-01
6	-2.630022647987E-02	1.020519885004E-01	4.747329886089E-01
6	2.630022647987E-02	-3.979480114996E-01	-4.747329886089E-01
1	-5.199622601196E-02	1.213225736934E-01	3.382879076928E-01
1	5.199622601196E-02	-3.786774263066E-01	-3.382879076928E-01
8	2.967856270488E-01	1.430573067445E-01	-4.697668277119E-01
8	-2.967856270488E-01	-3.569426932555E-01	4.697668277119E-01
6	-1.650551816042E-01	1.293087200599E-03	4.793691506985E-01
6	1.650551816042E-01	-4.987069127994E-01	-4.793691506985E-01
1	-1.505672468630E-01	-9.403825346480E-03	-3.856995046409E-01
1	1.505672468630E-01	4.905961746535E-01	3.856995046409E-01
8	-6.577788894868E-02	2.148708956961E-01	-4.506873074167E-01
8	6.577788894868E-02	-2.851291043039E-01	4.506873074167E-01
8	-1.266516189843E-01	-1.171560628906E-01	4.153864859241E-01
8	1.266516189843E-01	3.828439371094E-01	-4.153864859241E-01
6	-3.626189951292E-01	4.049078046134E-02	3.634633013087E-01
6	3.626189951292E-01	-4.595092195387E-01	-3.634633013087E-01
1	-3.705042486331E-01	1.452940543816E-01	3.566688891597E-01
1	3.705042486331E-01	-3.547059456184E-01	-3.566688891597E-01
1	-3.958995912826E-01	2.972490471667E-03	2.327826740244E-01
1	3.958995912826E-01	-4.970275095283E-01	-2.327826740244E-01
8	-4.950346209243E-01	-6.190444716624E-03	4.241416664773E-01
8	4.950346209243E-01	4.938095552834E-01	-4.241416664773E-01
1	-3.601614749440E-01	-2.999656489707E-01	3.662526460468E-01
1	3.601614749440E-01	2.000343510293E-01	-3.662526460468E-01
1	4.604890904021E-01	-1.018745756930E-01	-4.454858571386E-01
1	-4.604890904021E-01	3.981254243070E-01	4.454858571386E-01
1	-4.423520199225E-01	-4.335789830913E-01	-4.537797261382E-01
1	4.423520199225E-01	6.642101690866E-02	4.537797261382E-01
1	4.293190826571E-01	2.930113971780E-01	-1.015215815915E-01
1	-4.293190826571E-01	-2.069886028220E-01	1.015215815915E-01
1	4.706008807930E-01	3.571535401358E-01	1.721488816756E-01
1	-4.706008807930E-01	-1.428464598642E-01	-1.721488816756E-01
1	2.521528805485E-01	-1.052930497790E-02	2.347274596119E-02
1	-2.521528805485E-01	4.894706950221E-01	-2.347274596119E-02

Cellulose III$_1$

```
CRYSTAL
1 0 0
P 1 21 1
7.59 10.34 4.34 101.12
42
6    -5.283932474243E-02    -3.823522245143E-01   -2.483623566496E-02
6     5.283932474243E-02     1.176477754857E-01    2.483623566496E-02
6    -1.955133870483E-01    -2.870585447973E-01   -1.826675283183E-01
6     1.955133870483E-01     2.129414552027E-01    1.826675283183E-01
6    -1.659963405812E-01    -1.506940336151E-01   -4.136148303053E-02
6     1.659963405812E-01     3.493059663849E-01    4.136148303053E-02
6     3.346093098807E-02    -1.122295034780E-01   -1.388980400247E-03
6    -3.346093098807E-02     3.877704965220E-01    1.388980400247E-03
6     1.592351330935E-01    -2.191101535560E-01    1.538614278353E-01
6    -1.592351330935E-01     2.808898464440E-01   -1.538614278353E-01
6     3.571082882202E-01    -1.867073531237E-01    1.777649545264E-01
6    -3.571082882202E-01     3.132926468763E-01   -1.777649545264E-01
8    -3.672017115187E-01    -3.377340714284E-01   -1.689289794516E-01
8     3.672017115187E-01     1.622659285716E-01    1.689289794516E-01
8    -2.812436518697E-01    -6.787480042362E-02   -2.506352430371E-01
8     2.812436518697E-01     4.321251995764E-01    2.506352430371E-01
8     7.407457304783E-02     1.555005412292E-03    1.882524313666E-01
8    -7.407457304783E-02    -4.984449945877E-01   -1.882524313666E-01
8     1.219445352725E-01    -3.326116301703E-01   -3.776386283669E-02
8    -1.219445352725E-01     1.673883698297E-01    3.776386283669E-02
8     4.604138082761E-01    -3.000164751669E-01    2.753972532752E-01
8    -4.604138082761E-01     1.999835248331E-01   -2.753972532752E-01
1    -6.235705275973E-02    -3.979695475158E-01    2.241868269217E-01
1     6.235705275973E-02     1.020304524842E-01   -2.241868269217E-01
1    -1.816526407737E-01    -2.800029533575E-01   -4.302922719094E-01
1     1.816526407737E-01     2.199970466425E-01    4.302922719094E-01
1    -2.015117452800E-01    -1.491377902878E-01    1.943669768022E-01
1     2.015117452800E-01     3.508622097122E-01   -1.943669768022E-01
1     6.281233579020E-02    -9.324574071032E-02   -2.368224860377E-01
1    -6.281233579020E-02     4.067542592897E-01    2.368224860377E-01
1     1.345163394866E-01    -2.410130354202E-01    3.918498110316E-01
1    -1.345163394866E-01     2.589869645798E-01   -3.918498110316E-01
1     3.958354011563E-01    -1.071620173527E-01    3.451195049970E-01
1    -3.958354011563E-01     3.928379826473E-01   -3.451195049970E-01
1     3.828184877661E-01    -1.543720993343E-01   -5.140557728092E-02
1    -3.828184877661E-01     3.456279006657E-01    5.140557728092E-02
1    -4.464750239496E-01    -3.227634212453E-01   -3.828473840734E-01
1     4.464750239496E-01     1.772365787547E-01    3.828473840734E-01
1    -2.779003517654E-01     1.931417176386E-02   -1.597344113624E-01
1     2.779003517654E-01    -4.806858282361E-01    1.597344113624E-01
1    -4.652988303917E-01    -3.192237816786E-01    1.109718640146E-01
```

1	4.652988303917E-01	1.807762183214E-01	-1.109718640146E-01
